# Cell-Free Systems: Ideal Platforms for Accelerating the Discovery and Production of Peptide-Based Antibiotics

**DOI:** 10.3390/ijms25169109

**Published:** 2024-08-22

**Authors:** Hyeongwoo Park, Haneul Jin, Dayeong Kim, Joongoo Lee

**Affiliations:** 1 School of Interdisciplinary Bioscience and Bioengineering (I-Bio), Pohang University of Science and Technology, Pohang 37673, Republic of Korea; hwdream2016@postech.ac.kr; 2 Department of Chemical Engineering, Pohang University of Science and Technology, Pohang 37673, Republic of Korea; jinhn@postech.ac.kr (H.J.); dayeongk@postech.ac.kr (D.K.)

**Keywords:** antimicrobial peptides, peptide-based antibiotics, cell-free systems, non-canonical amino acids, ribosomally produced and post-translationally modified peptides, non-ribosomal peptides

## Abstract

Peptide-based antibiotics (PBAs), including antimicrobial peptides (AMPs) and their synthetic mimics, have received significant interest due to their diverse and unique bioactivities. The integration of high-throughput sequencing and bioinformatics tools has dramatically enhanced the discovery of enzymes, allowing researchers to identify specific genes and metabolic pathways responsible for producing novel PBAs more precisely. Cell-free systems (CFSs) that allow precise control over transcription and translation in vitro are being adapted, which accelerate the identification, characterization, selection, and production of novel PBAs. Furthermore, these platforms offer an ideal solution for overcoming the limitations of small-molecule antibiotics, which often lack efficacy against a broad spectrum of pathogens and contribute to the development of antibiotic resistance. In this review, we highlight recent examples of how CFSs streamline these processes while expanding our ability to access new antimicrobial agents that are effective against antibiotic-resistant infections.

## 1. Introduction

In an era when antibiotic resistance is rapidly becoming one of the greatest threats to global health, the race to discover and develop new antimicrobial agents has never been more urgent [1]. Recently, the World Health Organization (WHO) issued a warning on the potential consequences of a “post-antibiotic era” [2,3,4] in which common infections and minor injuries that have been treatable for decades could become dangerous. This alarming scenario has reignited interest in alternative therapeutic strategies, particularly PBAs, which show promise due to their potential to overcome traditional resistance pathways.

This renewed focus on novel antibiotics recalls one of the most monumental moments in medical history, the discovery of penicillin produced by the fungus *Penicillium notatum* in 1928, which revolutionized medicine by providing an effective treatment against bacterial infections. Structurally, penicillin contains a cyclic peptide (beta-lactam) backbone that is derived from a tripeptide scaffold, δ-L-(α-aminoadipoyl)-L-cysteinyl-D-valine [5,6,7]. This underscores that PBAs, including AMPs and their synthetic variants, can offer potent activity against resistant pathogens through mechanisms that differ from those of traditional antibiotics. Notably, the ability to incorporate monomers in addition to the 20 canonical amino acids with a repetitive polymeric backbone provides significant diversity, yielding products with unique mechanisms of action, effectiveness against multidrug resistant pathogens, and a low likelihood of cross-resistance [8]. As a result, this versatility enables them to target various cellular components, including membranes [9,10,11,12,13], biomolecules (enzymes [14,15], nucleic acids [16,17], and metabolites [18]), often with greater efficiency than small-molecule antibiotics (Table 1).

Recent cutting-edge technologies in high-throughput DNA sequencing and artificial intelligence (AI)-based bioinformatics tools have identified unique natural product gene clusters [38,39,40]. Continued exploration for PBAs using these techniques has also increased the possibility of discovering novel antibiotics with enhanced efficacy and reduced resistance [41,42]. A recently updated database, the Antimicrobial Peptide Database (APD3), which lists over 3000 PBAs from diverse natural sources (https://aps.unmc.edu, accessed on 1 February 2024), suggests an untapped potential for further discovery of novel bioactive materials. Despite their potential, the conventional methods for discovering and producing PBAs are often slow and limited by the complexities of production systems, such as isolation from natural sources or chemical synthesis of their derivatives. This limitation is evident in the fact that only 12 new antibiotics have entered the market from 2017 to 2021 [43]. In the 2021 WHO annual analysis, only 27 new antibiotics were in clinical trials against critical pathogens [4], and of these 27, only 6 were considered to be capable of overcoming antibiotic resistance. In contrast, more than 1300 cancer drugs were in clinical trials in 2020 [44]. This is why CFSs have emerged as a groundbreaking platform, offering unprecedented speed, flexibility, and efficiency in the discovery and synthesis of PBAs.

Unlike traditional in vivo systems, CFSs mediate protein translation via the ribosome in a controlled, open environment, enabling rapid and flexible experimentation [45]. As researchers continue to explore the full potential of CFSs [46,47], these systems have become poised to play a pivotal role in addressing challenges of rapid discovery, design, screening, and biomanufacturing of medically important peptides [48,49,50,51]. While CFSs have been extensively used for research on peptides, proteins, and enzymes, reviews focusing on PBAs integrated with CFSs are lacking. In this review, we begin by categorizing natural PBAs (Section 2) and providing a brief overview of CFSs (Section 3). Next, we present key advancements in CFSs used for the identification of mechanisms of action of PBAs (Section 4.1), characterization of biosynthetic pathways (Section 4.2), high-throughput screening (Section 4.3), and direct, rapid synthesis of PBAs with complex structures (Section 4.4). Overall, CFSs are powerful tools with the potential to rapidly create and diversify PBAs, bridging gaps in the traditional technologies used for the identification, characterization, and production of antibiotics.

## 2. PBAs Are Produced through Various Biosynthetic Pathways

Based on the molecular mechanisms involved in the synthesis of bioactive peptides in microorganisms [9,52,53,54], we categorize them in two broad groups: ribosomally (Group I) or non-ribosomally (Group II) produced peptides (Table 2). Ribosomally produced peptides can be further divided into two different subgroups. The first subgroup of Group I, shown in Table 2(a), is produced by the direct polymerization of the 20 canonical amino acid monomers via the translational machinery (aminoacyl tRNA synthetases (aaRSs), elongation factors, and ribosome). These PBAs can be rapidly designed and synthesized both in vivo and in vitro because they are directly synthesized by the ribosome using the cell’s genetic code in messenger RNA (mRNA) as a template [55,56,57,58]. This allows for the efficient production of diverse variants that may selectively target one or multiple pathogenic bacteria [59,60,61,62], thereby making them promising candidates for therapeutic applications [63].

The folded states of such PBAs fall into three broad structural categories: α-helical peptides (e.g., LL37, melittin) [9,64]; extended structures abundant in proline (Api-137) [63,65], tryptophan (indolicidin) [66], or arginine (bactenecin) [67]; and macrocyclic loops with one or multiple disulfide bonds (SFTI-1, kalata B1) [68,69,70]. In general, α-helical peptides disrupt cell membranes. These helical structures provide amphipathic properties, allowing them to insert into microbial cell membranes effectively and destabilize the lipid bilayers [71,72], ultimately causing cell lysis and death. Compared to α-helical peptides, extended structures rich in specific amino acids (e.g., Pro, Trp, and Arg) utilize their unique molecular characteristics. For example, proline-rich AMPs (PrAMPs) adopt a rigid conformation that facilitates membrane penetration and intracellular targeting with ribosomes, leading to inhibition of protein synthesis [73,74]. Tryptophan-rich AMPs (WrAMPs) leverage their aromatic side chains for strong membrane interactions, leading to pore formation in the membrane. Arginine-rich peptides (RrAMPs) exploit their positive charge to interact with membranes and nucleic acids, disrupting essential cellular processes. The cyclic architectures formed by disulfide bonds create a rigid structure, resulting in high-affinity interactions with biological targets due to reduced entropic effects and resistance to proteolytic degradation [75,76,77,78,79,80].

**Table 2:** Classification of peptide-based antibiotics (PBAs).

Group	Machinery for Biosynthesis	Types of Peptides	Representative Compounds
I	(a) Translational apparatus	Peptides only containing the 20 canonical amino acids	α-Helical peptides [9], PrAMPs [63,65], WrAMPs [66], RrAMPs [67], disulfide-rich peptides [69,70]
	(b) Translational apparatus and post-translational enzymes	Ribosomally synthesized and post-translationally modified peptides (RiPPs)	Linear architecture (azole-containing peptides (LAPs) [81], cyanobactin [82], linaridin [83]) Macrocyclic architecture (lanthipeptides [84], thiopeptide [85], lasso peptides [86])
II	(c) Non-ribosomal peptide synthetases (NRPSs)	Non-ribosomal peptides (NRPs)	Lipopeptide surfactants [87], cyclodecapeptides [88], glycopeptide antibiotics [89], mycobactins [90], malleobactins [91]

The second subgroup of Group I, shown in Table 2(b), consists of PBAs that contain one or multiple non-canonical amino acids (ncAAs), thereby acquiring novel bioactivities. A representative example is ribosomally synthesized and post-translationally modified peptides (RiPPs). They differ from the first subgroup in that they have diverse structures beyond the linear peptide backbone and include more than the 20 canonical amino acids. This structural diversity arises from various post-translational modifications (PTMs), such as cyclization, cyclodehydration (ring-closing condensation), glycosylation, N-alkylation, and others (Table 3). Cyclization is a key modification in the production of lanthipeptides and lasso peptides [86,92], involving mechanisms such as thioether bridge formation, cysteine reduction, or head-to-tail cyclization. Cyclodehydration is another crucial process, forming biologically active motifs by producing heterocycles such as thiazoles and oxazoles through the dehydration of serine, threonine, or cysteine residues and subsequent ring-closing condensation with the peptide backbone [93,94,95]. Glycosylation is a significant PTM catalyzed by various glycosyltransferases that attach carbohydrate moieties (glycans or oligosaccharides) to specific amino acid residues within ribosomally produced peptides. This modification enhances the structural stability, solubility, and bioactivity of RiPPs [96]. N-alkylation, most commonly N-methylation, is catalyzed by N-methyltransferases, which transfer a methyl group from a donor molecule, typically S-adenosylmethionine (SAM), to the nitrogen atoms of amino acid residues within peptides [97,98]. Methylation helps maintain the structural integrity of peptides under various stress conditions, such as changes in pH or temperature [99].

**Figure 1:**
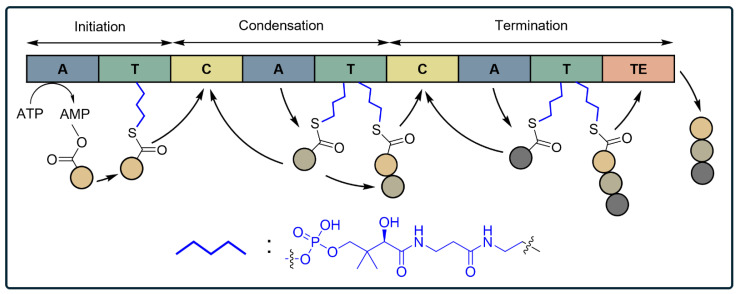
NRPSs are large complexes composed of adenylation (A), thiolation (T), and condensation (C) domains. Non-ribosomal peptides are synthesized through sequential enzymatic reactions: the A domain activates and attaches amino acids (colored circles) to the T domain, initiating biosynthesis by forming a thioester bond with Ppant (blue). The C domain then forms peptide bonds between modules. During termination, the thioesterase (TE) domain hydrolyzes the bond between the T domain and the peptide, releasing the final peptide product.

Group II PBAs, shown in Table 2(c), include non-ribosomally synthesized peptides produced through a complex enzymatic process that operates independently of the translational apparatus and mRNA templates [52,100,101,102]. This process is mediated by non-ribosomal peptide synthetases (NRPSs), which are large, multi-modular enzyme complexes that function like an assembly line to incorporate a wide variety of substrates. The modules typically include domains for adenylation (A), thiolation (T), condensation (C), and thioesterase (TE) [98]. In the A domain, the enzyme selects and activates specific amino acids by forming an aminoacyl-adenylate intermediate. The activated amino acid is then transferred to the T domain, where it is covalently attached to a 4′-phosphopantetheine (Ppant) prosthetic group, creating a thioester bond. This thioester linkage secures the growing peptide chain to the enzyme, allowing it to be carried to the C domain. In the C domain, the enzyme catalyzes the peptide bond formation between the incoming amino acid and the nascent peptide chain. The TE domain is crucial for releasing the completed peptide from the NRPS complex. It catalyzes the cyclization or hydrolysis of the thioester bond, thus liberating the final peptide product. This sequential addition and elongation process ensures that amino acids are added in a specific and ordered manner, resulting in the precise assembly of the peptide product (Figure 1) [98,103]. Due to this characteristic, NRPS allows the incorporation of diverse non-canonical substrates, not being limited to the 20 standard amino acids, producing peptides with unique structures and biochemical properties, including increased stability, enhanced biological activity, and resistance to enzymatic degradation [104]. Various non-canonical substrates incorporated by NRPS include D-amino acids [105], hydroxyacids [106], N-alkylated amino acids [107], β-amino acids [108], and others [109]. Subsequent chemo-enzymatic modifications of side chains after peptide synthesis result in even more unique structures, such as cyclic [110] and branched [111] structures, or with multiple rings and cross-links [112]. These attributes make NRPs effective and selective in their biological activities, often offering antimicrobial, anticancer, and immunosuppressive properties [113,114]. Such versatility and robustness highlight the potential of NRPs in therapeutic applications and drug development.

An ideal platform for the development of PBAs with the characteristics discussed above would be a comprehensive system that integrates several key functions essential for efficient and effective discovery and production. First, it would facilitate the identification of the mechanism of action of PBA. This involves advanced tools such as high-resolution imaging [9], biochemical assays [115], and computational modeling [116] to understand how PBAs interact with bacterial targets at the molecular level. By elucidating the specific interactions and pathways through which PBAs exert their antimicrobial effects, researchers can pinpoint the sequences responsible for efficacy and optimize the composition of PBAs for enhanced activity. Second, the platform would enable the elucidation of biosynthetic pathways. Understanding these pathways is critical for revealing the step-by-step process by which these peptides are assembled, modified, and matured, providing insights into how to manipulate and optimize these pathways for better yield and functionality. Third, the system would allow for the selection of efficient candidates from a library. This requires a robust method for screening a vast array of potential peptide sequences to identify those with the most desirable properties, such as high antimicrobial activity, stability, and low toxicity. High-throughput screening techniques and machine learning algorithms could play a significant role in this selection process. Finally, the platform should support the rapid production of complex structures. This involves the ability to synthesize these peptides quickly and accurately, both in vitro and in vivo, using non-canonical substrates. Cell-free systems exemplify such an ideal platform, offering the flexibility and efficiency needed for each of these stages, from identification to production of diverse PBAs.

## 3. CFSs as Ideal Platforms for PBA Research

**Figure 2:**
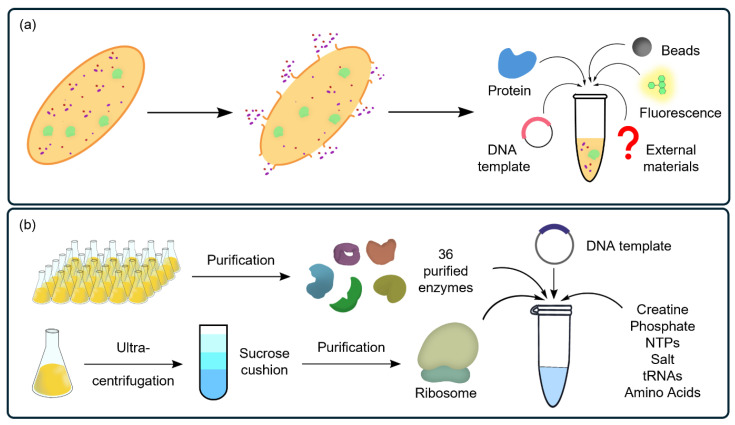
Two types of cell-free systems (CFSs). (**a**) Lysate-based CFSs (yellow) provide a cell-like environment. (**b**) Reconstituted CFSs (blue) contain a minimal set of 36 purified enzymes required for transcription and translation for protein synthesis. The open environment of CFSs allows the precise control of biosynthetic pathways of PBAs through the modular assembly of required components.

CFSs provide ideal platforms as they meet the essential requirements for studying PBAs. The platforms have two main types: lysate-based and reconstituted systems, which offer distinct advantages [45]. Briefly, lysate-based systems, as shown in Figure 2a, are derived from cell extracts obtained through relatively simple methods, such as sonication [117,118], high-pressure homogenization [119,120], and enzymatic lysis [121]. These systems are typically produced using prokaryotic cells, such as *Escherichia coli* (*E. coli*) [122,123] or other bacterial cells [124,125]. The minimalistic nature of prokaryotic cells, which lack complex organelles and extensive PTM pathways, simplifies the synthesis of target proteins with high productivity (~g/L/h) [126,127]. In contrast, PTM pathways in eukaryotic cells involve a series of enzymatic reactions that modify proteins after translation, enabling them to become fully functional. These modifications include glycosylation [119], phosphorylation [128], ubiquitination [129,130], methylation [131], acetylation [132], and the formation of disulfide bonds [133], among others. Each of these modifications requires specific enzymes and cellular machinery, such as the endoplasmic reticulum and Golgi apparatus. Therefore, eukaryotic cell-free systems provide an optimal balance between the simplicity of prokaryotic expression systems and the complexity of whole-cell eukaryotic systems by harnessing the cellular machinery required for PTMs and protein folding without the need to maintain living cells. Eukaryotic cell-free systems use extracts from yeast [134,135], wheat germ [136], or Chinese hamster ovary (CHO) cells containing the enzymes and molecular chaperones required for performing PTMs and ensuring correct protein folding [137]. This enables the production of high-value proteins, such as therapeutic antibodies that require precise folding and glycosylation, to function effectively. 

Reconstituted systems (Figure 2b) consist of a defined set of purified components that are essential for transcription and translation [138]. These typically include ribosomes, tRNAs, aaRS, nucleoside triphosphates (NTPs), and translation factors for initiation, elongation, and termination. These systems offer a high degree of control over the protein synthesis process by using individually purified components, which allows researchers to eliminate undesired biochemical reactions associated with crude extracts, such as proteolysis [139] and nucleic acid degradation [140]. The PURExpress^®^ (New England Biolabs) system is one of the most well-known commercially available reconstituted CFSs [141]. Recently, methods to construct reconstituted systems have been developed, enabling researchers to efficiently produce their in-house systems in the laboratory. Due to this, reconstituted CFSs are often an ideal choice for research on PBAs. Gabant et al. synthesized multiple active bacteriocins in parallel by using a standardized synthetic gene library in PURExpress^®^, demonstrating its potential for the direct, fast, and accurate synthesis of active PBAs [142]. The flexible in vitro translation (FIT) system developed by Hiroaki Suga exemplifies a reconstituted system that enables the synthesis of non-standard peptides by incorporating ncAAs through a reprogrammed ribosomal translation process [143]. Genetic code reprogramming is achieved by a ribozyme, flexizyme, which creates misacylated-tRNAs with ncAAs. Flexizyme facilitates the attachment of a wide range of ncAAs to tRNAs, enabling their incorporation into peptides during in vitro translation [50,144,145]. This technology greatly expands the potential of peptide synthesis, allowing for the production of novel PBAs with unique properties [146]. Another key factor of the FIT system is its use of open reaction characteristics of CFSs. For PBA synthesis, additional enzymes are supplemented in the reaction, which enhances the biosynthetic process or PTMs in vitro. 

However, reconstituted systems are relatively difficult to construct compared to lysate-based systems, due to the extensive purification steps required for the minimal components. Importantly, maintaining the stability and activity of these purified components can be challenging, further complicating the construction and operation of reconstituted systems.

## 4. Main Research on PBAs Using CFSs

Utilizing the benefits of CFSs for PBA studies, researchers have made significant efforts in four broad areas: understanding the molecular mechanisms of antimicrobial activity, elucidating biosynthetic pathways, developing new methods for selection, and enabling large-scale synthesis of various PBAs (Table 4) [147]. In our review, we categorized the four major areas of PBA mentioned above into separate sections, introduced the corresponding research in each, and focused on summarizing those studies (Table 5).

### 4.1. CFSs Enable the Elucidation of the Mechanism of Action of PBAs 

PBAs combat bacterial infections through various mechanisms (Table 1). These mechanisms include cell membrane disruption [173], pore formation [174], inhibition of protein synthesis [175], interference with nucleic acid functions [176], immune modulation [177], biofilm disruption [178], and reactive oxygen species production [179]. Mechanistic studies aim to understand how PBAs exert their effects at the molecular level, which in turn provides insight for designing PBAs with enhanced specificity and altered toxicity. Thus, various methods combining biochemical [9,180], biophysical [181], and molecular biology [182,183] techniques have been developed. Advanced techniques such as spectroscopy (nuclear magnetic resonance or circular dichroism) [184,185] and microscopy (electron microscopy or confocal fluorescence microscopy) [9,156] reveal structural details of peptide interactions with membranes and intracellular targets.

**Figure 3:**
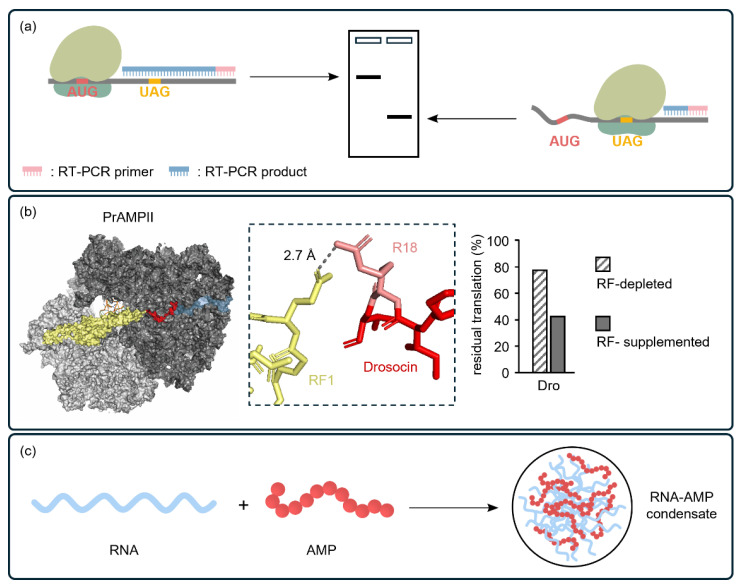
Cell-free systems as ideal platforms for elucidating the intracellular mechanisms of the antimicrobial action of PBAs. (**a**) In a reconstituted cell-free system, deleterious enzymes (e.g., RNases, DNases, and proteases) that could reduce protein production are excluded. This allows for the use or synthesis of short DNAs, RNAs, or peptides. The toe-printing technique, which utilizes ribosome stalling, helps identify the PBA mechanisms that inhibit ribosome polymerization. The platform enables the identification of inhibited translation stages in the discovery process, especially if the PBAs target the large subunit of ribosomes. (**b**) The inhibition mechanism of type II PrAMPs (drosocin) is elucidated on a cell-free platform by selectively adding or removing RF1. Cryo-EM analysis shows that drosocin binds to the exit tunnel of the ribosome and interacts with RF1 located in the A-site (exit tunnel of the ribosome: blue, drosocin: red, RF1: yellow), inhibiting translation efficiency by approximately 50%. (**c**) The capability to form biomolecular condensates (RNA and PBA) via phase transitions is the origin of bactericidal activity.

Recent studies using CFSs have contributed to a better understanding of these mechanisms of action. Toe-printing assays (Figure 3a), also known as primer extension inhibition assays, are applied in vitro to study the interactions between PBAs and the ribosomes during protein synthesis [186,187]. In these assays, a chemical probe-labeled primer is extended by reverse transcriptase until it encounters a ribosome stalled on the mRNA, resulting in a “toe-print” that indicates the position of the ribosome. This technique provides detailed and direct insights into how PBAs affect the intracellular translation reaction at specific sites along the mRNA [188]. A key to success was using CFSs in which enzymes (e.g., DNases, RNases, and proteases) or other cellular components unrelated to toe-printing that could interfere with ribosome stalling [156,189,190] were selectively eliminated. Similarly, Koller et al. further demonstrate how O-glycosylated drosocins (type II PrAMPs) enhance their antimicrobial activity (Figure 3b) at the molecular level using a CFS [156]. They revealed that type II glycosylated drosocins do not disrupt the cellular membrane, but instead enter the intracellular space and arrest ribosomes at stop codons by binding to the ribosome’s exit tunnel and release factor 1 (RF1) in the A-site. This insight helped them understand the mechanism of inhibition: the binding of drosocin to the ribosome and RFs deplete free RFs in the cellular environment, preventing the ribosomes from releasing completed proteins at stop codons, thereby causing ribosome stalling and misincorporation or frameshifting.

Another recently elucidated novel mechanism involves PBAs targeting the phase transitions of cytosolic nucleic acids. Using machine learning sequence analysis, Sneideris et al. identified new PBAs that readily form liquid-like condensates with nucleic acids through phase separation (Figure 3c) [16]. These PBAs often include charged and polar amino acid residues, which interact strongly with nucleic acids. This results in the sequestration and compaction of nucleic acids in living cells, ultimately leading to bacterial cell death. Notably, this capability correlates with their inhibition of prokaryotic transcription and translation, as well as their interaction with bacterial nucleic acids in vivo, suggesting that designing PBAs capable of forming condensates could be a key to discovering new PBAs.

### 4.2. CFSs Facilitate the Characterization of Biosynthetic Pathways of PBA

Identifying biosynthetic gene clusters (BGCs) is also a crucial step in the discovery of new peptides, which can inform strategies for PBA production using enzymes. Recent studies have employed genome mining and bioinformatics tools to identify BGCs that encode target PBAs [62,191,192]. Investigating BGCs using CFSs provides a comprehensive framework for studying biosynthetic pathways [149,150,151] (Table 4), as each step of the biosynthesis can be independently controlled with a specific enzyme in separate reactions. This approach allows for the exploration of enzyme promiscuity [157,158,159,160,161,162,163,171,172] and kinetics [152] required for each biosynthetic step. Importantly, by using this method, the range of substrate analogs for an enzyme can be determined and enzymes can be further engineered with mutations at catalytic sites. This ultimately contributes to the discovery of novel bioactive compounds and the diversification of products that might be inaccessible through in vivo methods.

**Figure 4:**
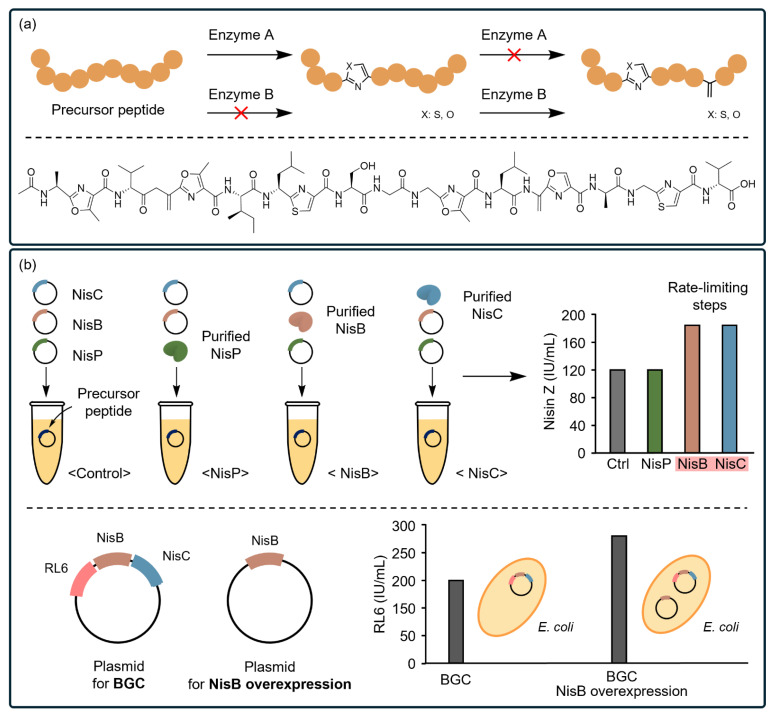
CFSs facilitate the characterization of biosynthetic pathways of PBAs. (**a**) The sequence of PTMs within a biosynthetic gene cluster (BGC) can be efficiently confirmed using a reconstituted CFS. Enzyme A and B of the BGC should react sequentially to produce an azole-ring and a dehydroalanine motif. Enzymatic reactions in reverse order do not yield the desired product (red x). Goadsporin (lower panel), an azole-containing linear PBA, is synthesized using a FIT system by supplementing the key PTM enzymes in a sequential order. (**b**) To analyze the rate-limiting step of NisZ biosynthesis mediated by NisP, NisB, and NisC, an excess amount of the purified enzyme involved in the targeted step is supplied to the in vitro reaction, while the other enzymes are expressed directly from plasmids at low concentrations. The increase in production indicates that the step catalyzed by the additionally supplied enzyme is rate limiting (upper panel). For large-scale synthesis, the key enzymes are overexpressed in plasmids, enabling *E. coli* to produce the target lanthipeptide analog (RL6) in large amounts (lower panel).

Thiopeptides, linear azole-containing peptides (LAPs), and lanthipeptides feature complex structures beyond simple linear peptide backbones. These structures are formed post-translationally by multiple enzymes encoded within their gene clusters [40]. These diverse classes of RiPPs contain cyclic motifs, such as thiazoles with macrocyclic (loop-like) structures, heterocyclic azolines, and thioether bonds [193]. Recently, Vinogradov et al. demonstrated the synthesis of a thiopeptide, known as lactazole A, using an FIT-Laz system. In this system, the key enzymes necessary to form the unique structures during the biosynthetic pathways are either directly produced from a DNA template or produced separately in vivo and supplemented as purified enzymes [157]. This approach enabled the identification of the minimal lactazole scaffold required for bioactivity. Similarly, a recent study identified the specific roles of enzymes required for intricate biosynthesis using a FIT platform, which synthesizes unique structural elements, such as an N-terminal acetyl moiety, azoles, and dehydroalanines (Figure 4a). By supplementing each enzyme in various combinations within a one-pot, cell-free protein synthesis system, the authors clarified the sequential order of modifications and facilitated the production of goadsporin analogs [158]. 

Building on the success of elucidating PTM reactions and discovering their cognate enzymes, CFSs have further demonstrated their potential for the overproduction of novel lanthipeptides [152]. Lanthipeptides are promising PBAs with significant therapeutic applications [131]; however, their production faces challenges at an industrial scale due to their toxicity to the host bacterial cells and the complexity of biosynthesis pathways. Recently, Liu et al. presented a novel CFS that enables the discovery and production of lanthipeptides (Figure 4b) [139]. The authors analyzed gene cluster through genome mining and identified essential enzymes (NisP, NisB, and NisC) that facilitate the bottleneck steps of biosynthetic pathways. Supplementation with the purified enzymes significantly increased the yield (180 IU/mL) of active lanthipeptides. By leveraging the enzymes’ promiscuity toward various substrates, they successfully discovered four novel lanthipeptide analogs that are not toxic to host cells. Based on these findings, they implemented the systems into host cells (*E. coli*) and produced the lanthipeptide analogs (260.1 ± 10.5 IU/mL).

### 4.3. CFSs Enable the Selection of PBAs from a Diverse Library

High throughput screening (HTS) is a highly efficient technique that allows researchers to quickly evaluate and identify potential PBAs from vast libraries of compounds. By automating the testing process, HTS enables the simultaneous analysis of thousands to millions of samples, significantly accelerating the discovery of novel PBAs with desired biological activities [133]. Building on these achievements, CFSs provide a cost-effective platform for screening large peptide libraries and enable the customization of peptide synthesis components, further enhancing the efficiency of the screening process [168,169]. 

**Figure 5:**
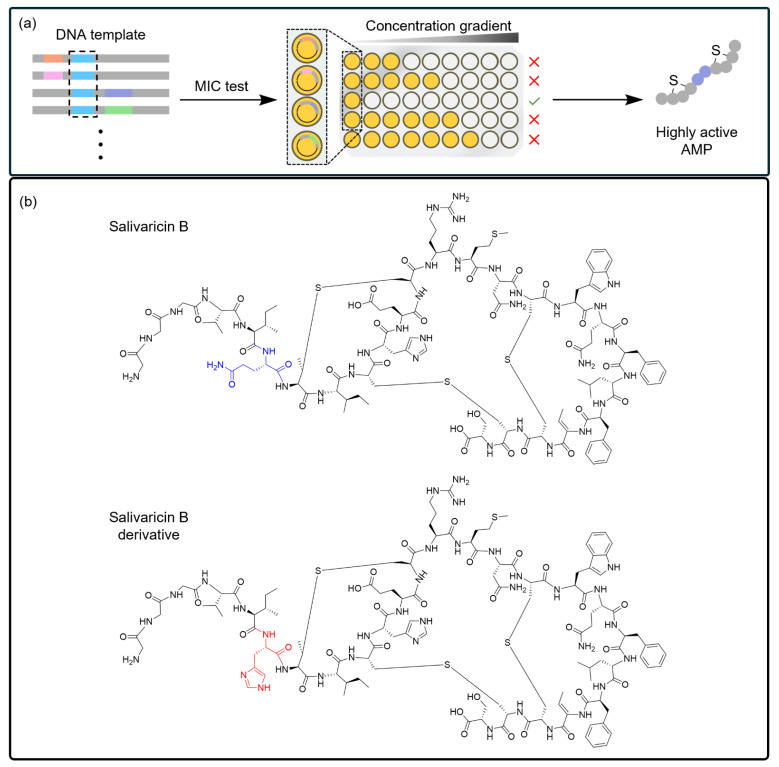
Screening for salivaricin B synthesis and its derivatives obtained from the selection process. (**a**) Genome mining and screening platforms enable the production of a library containing mutations at multiple sites of the wild-type salivaricin B structure. The enzyme-binding sites (light blue) revealed via genome mining remained intact during mutagenesis. Using cell-free protein synthesis platforms, a novel salivaricin B derivative with high antimicrobial activity (green √) against gram-negative bacteria was discovered. (**b**) Through a selection process from a library containing numerous derivatives, a new salivaricin B derivative with 4-fold enhanced efficacy was found to contain a histidine residue (red).

In the study on thiopeptide analogs discussed in the previous section, the authors further demonstrated the capability of the CFS to select the most efficient lanthipeptides from a library. In a new CFS, in which the genes encoding proteases or peptidases from the *E. coli* genome were removed, the authors produced ~3000 highly active variants of the salivaricin B gene [139] (Figure 5a). Among these, a salivaricin B mutant with 4-fold higher antibacterial activity than salivaricin B against *B. subtilis*, *M. luteus*, and *L. lactis* (Figure 5b) was identified through minimum inhibitory concentration (MIC) testing (1 µM, 0.5 µM, and 1 µM, respectively) [139]. These findings highlight the broad applicability of the CFS platform for selecting new variants with enhanced antimicrobial activity.

**Figure 6:**
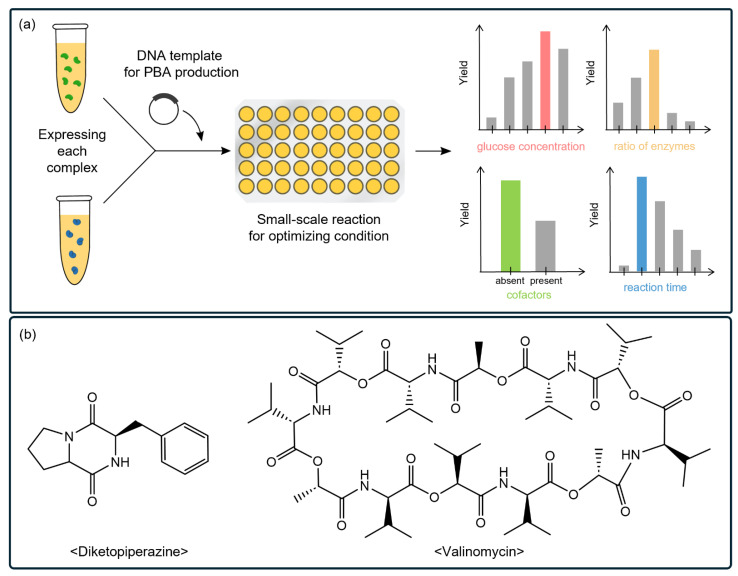
CFSs for screening targeted PBAs. (**a**) Biosynthesis of NRPs using NRPSs requires precise reaction conditions to achieve high yields. NRPS modules are produced separately and combined into a multi-well plate to identify optimal reaction conditions, such as glucose concentration, NRPS ratios, cofactors (NAD, CoA, and ATP), and reaction time. (**b**) Molecular structures of diketopiperazine and valinomycin are produced efficiently by optimizing synthetic conditions using a CFS.

HTS assays also help pinpoint optimal reaction conditions for high-yield production [164,165,194]. Recent studies have demonstrated that CFSs can be used to reconstruct large NRPSs and directly produce natural products using template DNA supplemented in the reaction [164,166]. The NRPS system typically requires customized reaction conditions, such as glucose concentration, enzyme ratios, cofactors, and reaction time (Figure 6a), which are essential for the optimal synthesis of target PBAs. The optimal conditions identified in the CFS enabled efficient NRPS synthesis, thereby facilitating the in situ production of natural products, such as diketopiperazine [165] and valinomycin [164] (Figure 6b). These results demonstrate the capability of CFSs to overcome the limitations of traditional cell-based expression systems that lack the flexibility to finely control the synthetic conditions.

**Figure 7:**
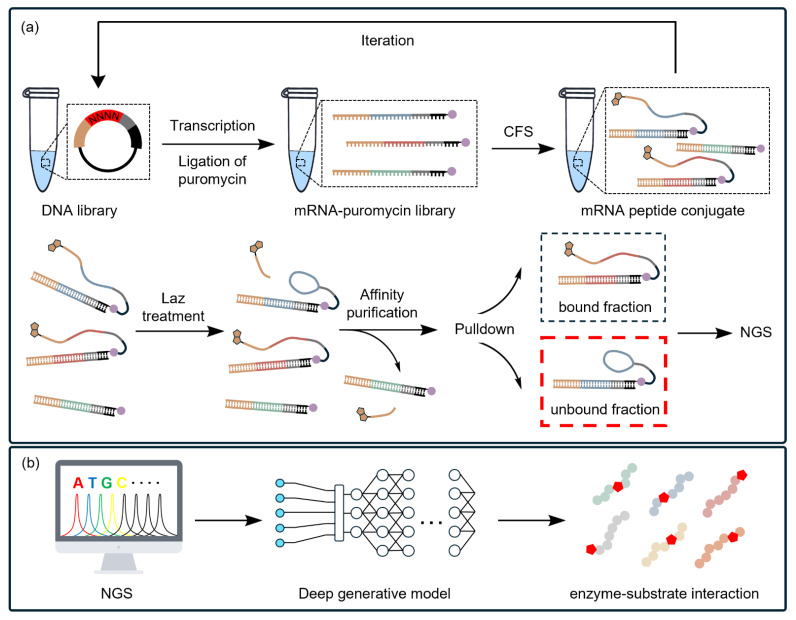
Schematic of the research for investigating lactazole BGCs. (**a**) For the reactivity-profiling mRNA display assay, N-terminally biotinylated thiopeptide precursors are produced using a CFS by reprogramming translation initiation. These biotinylated peptides are then displayed on mRNAs, forming peptide–mRNA conjugates by the nucleophilic attack of the amino group of puromycin (purple circle) to the nascent peptide chain in the ribosome. During the assay, the conjugates are treated with a set of Laz enzymes, which convert some precursors to mature thiopeptides by cleaving off the biotin tag. A streptavidin-based pulldown step is then used to remove the reactive (biotin-cleaved, mature, black dotted box) from the unreactive (biotin-retained, only partially modified or not modified) fractions (red dotted box). (**b**) Peptide sequences are scored using the “Y score”, which is determined by analyzing the frequency of each peptide in both fractions of the library. This model predicts the promiscuity of lactazole RiPP enzymes, facilitating the de novo design of thiopeptide derivatives bearing a thiazole (red pentagon).

Returning to the identification of the minimal lactazole (a type of RiPP) scaffold discussed in the previous section, Vinogradov et al. further explored expanding the range of substrates acceptable for the enzymes used in lactazole biosynthesis [171,172]. Using a reactivity-profiling mRNA display assay, which establishes a genotype–phenotype link through mRNA display techniques and indirectly identifies amino acid sequences via sequencing the conjugated mRNA [171], the authors identified alternative substrate preferences in the biosynthetic pathway (Figure 7a). Subsequently, amino acid sequences scored by next generation sequencing (NGS) were used to train a deep generative model that generated a diverse array of lactazole-forming sequences based on minimal lactazole scaffolds. This assay not only confirmed known aspects of lactazole biosynthesis but also provided new insights into enzyme fitness, enabling the construction of combinatorial thiopeptides, and leveraging the capabilities of CFSs for high-throughput analysis. The model also predicted the promiscuity of lactazole RiPP enzymes, aiding in the design of thiopeptide derivatives (Figure 7b).

**Figure 8:**
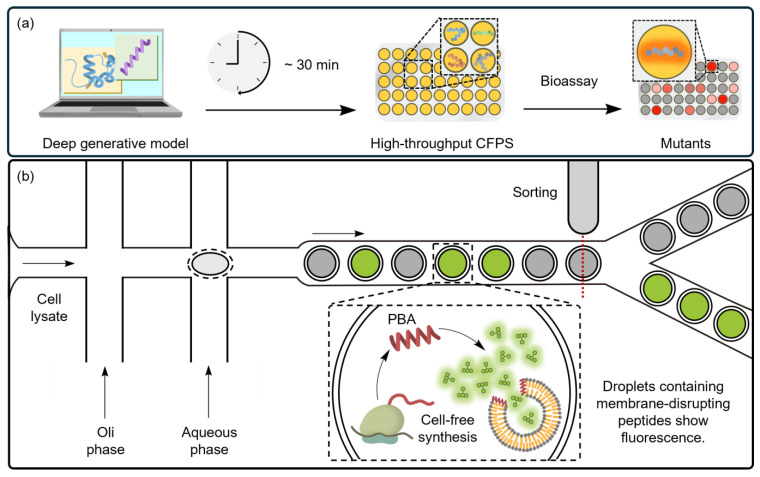
Diverse platforms using CFSs for screening PBAs. (**a**) To design PBAs de novo for new drug discovery, a deep generative model is integrated with a CFS. The model prioritizes potential PBAs and predicts target PBAs with higher accuracy. In the experimental pipeline, PBAs are rapidly synthesized from a synthetic DNA template in the CFS and directly tested against bacterial cultures. Notably, the six newly discovered de novo-designed PBAs exhibit antimicrobial activity against a wide spectrum of multidrug-resistant pathogens. (**b**) Microfluidic systems that generate multiple droplets containing different PBAs provide an efficient way to identify new PBAs with high membrane disruption activity. A self-quenching dye is embedded in a vesicle, which is further encapsulated in bacteria-like lipid vesicles. Increased fluorescence indicates that the PBA disrupts the inner vesicle, resulting in the dilution of the self-quenched dye within the droplet.

Combined with CFSs, deep learning models have significantly expanded our ability to discover new PBAs [195,196]. These models typically learn from existing PBAs to propose novel protein sequences, enabling the creation of thousands of new designs much faster than traditional methods. A recent study showed that this platform could generate multiple de novo PBAs from synthetic DNA fragments within 30 min, followed by direct minimum inhibitory concentration (MIC) testing of these PBA mutants within the CFS (Figure 8a) [168]. This integration further accelerates the discovery and validation of new antimicrobial agents at a low cost.

Microfluidic systems also play a crucial role in accelerating the generation and selection of PBAs for high-throughput screening. Vesicle structures with lipid membranes have shown the ability to screen PBAs based on membrane specificity. Nuti et al. demonstrated that double emulsion droplets can be generated at high frequency by microfluidic systems for PBA selection (Figure 8b) [170]. In this system, diverse PBAs are synthesized within large vesicles that co-encapsulate smaller droplets containing self-quenched fluorescent dyes. When PBAs disrupt the membranes, the dye is released, producing a fluorescent signal. These fluorescent droplets are then sorted, and the DNA template within each droplet is sequenced for PBA identification.

### 4.4. CFSs Enable the Synthesis of PBAs with Complex Structures

PBAs found in nature often have complex molecular structures that enhance their ability to bind accurately to targets [113], increasing their effectiveness at low concentrations and reducing the risk of off-target effects [114]. These intricate structures also provide significant stability, making PBAs highly resistant to proteolytic degradation in cellular environments due to their unique topological features, similar to the stability observed in macrocyclic structures [197,198]. In addition, the process of stapling residues can further improve the bioavailability of PBAs by increasing their structural rigidity and locking the peptides into active conformations, thereby resulting in higher affinities for their targets [199,200].

**Figure 9:**
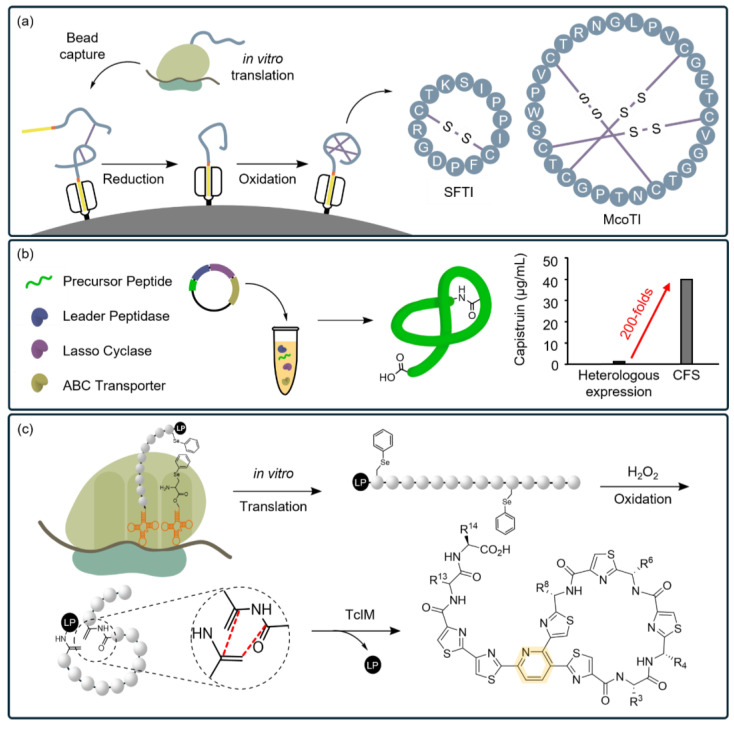
CFSs enable the synthesis of more complex protein structures. (**a**) Resin-assisted reduction and oxidative folding. Beads, utilized to capture the tag of disulfide-rich PBAs, are supplied into a CFS to avoid the trade-off between folding and aggregation, sequestering both on-path and off-path folding intermediates. Following the capture of the tag, disulfide bonds are completely reduced. Subsequently, the oxidation of cysteine is reperformed under thermodynamic control, enabling the reshuffling of disulfide bonds. This process ensures that PBAs adopt the most stable and functional structure via multiple disulfide bonds. (**b**) A co-expression of enzymes required to process precursor peptides significantly increases the yield of lasso peptides by up to 200-fold compared with conventional heterologous expression strategies. (**c**) The open environment of the reconstituted CFS allows for the incorporation and in vitro translation of ncMs, followed by additional in situ chemo-enzymatic reactions. By excluding specific aaRSs or RFs, ncM is introduced into the peptide without competition from the endogenous tRNAs. Chemo-enzymatic reactions between non-canonical motifs incorporated into a peptide form a novel architecture (e.g., cyclic peptide), expanding the diversity of the PBAs produced through CFSs.

The most common covalent bond providing structural stability in nature is the disulfide bond formed between the thiol groups of cysteine residues [78]. Wu et al. recently introduced a method to improve the folding and reduce the aggregation of disulfide-rich peptides and proteins produced in vitro (Figure 9a) [153]. They used a modified *E. coli*-based CFS to enable the co-translational capture of peptides using an affinity matrix. This facilitated accurate oxidative folding through disulfide bonds and recycling of misfolded states. This approach enhances the yield (4~5-fold) of peptides with complex macrocyclic architectures and multiple disulfide bonds, indicating that CFSs could be applied to various peptides that need to maintain their structure under denaturing conditions, such as high temperature, acids, or bases.

Lasso peptides are a prevalent class of RiPPs produced in bacteria (e.g., *E. coli*, *B. caledonica*, *S. aureus*, *B. subtilis*, and *P. aeruginosa*) [86,201,202]. Their lariat-like threaded conformation imparts unique anticancer [203] and antibacterial activities [204], along with resistance to thermal and proteolytic degradation [202]. Despite these significant advantages, the production efficiency of lasso peptides is low due to the lack of an appropriate synthesis platform [205,206]. Understanding the BGCs of these peptides is essential for identifying the regions critical to their unique conformation, which is key to discovering and synthesizing exciting structures [86]. Si et al. recently demonstrated that a wide variety of lasso peptides could be synthesized using a CFS based on the rational design of precursor substrates containing a leader sequence. The identification of a promiscuous enzyme, lasso cyclase, was another critical factor in facilitating lasso formation (Figure 9b) [154]. Using a CFS, they achieved a 200-fold increase in production efficiency compared to traditional heterologous expression methods.

A CFS developed by Fleming et al. for thiopeptide synthesis demonstrates the potential for integrating additional chemo-enzymatic reactions and ncAAs to synthesize even more complex structures [167]. This CFS enables the incorporation of a selenocysteine residue, which undergoes oxidative elimination with hydrogen peroxide to generate dehydroalanines (Figure 9c). The addition of a PTM enzyme, TclM, facilitates an enzymatic cycloaddition reaction between the dehydroalanines, converting a linear structure into a lasso structure featuring a six-membered heterocyclic pyridine motif.

**Table 5:** The list of CFS-mediated PBAs covered in this article.

Section	PBAs	Structural Features	Target Microorganisms
Section 4.1	Drosocin	O-glycosylation	Gram-negative bacteria (*E. coli*)
	Os-C	-	Bacteria
	P113	C-terminus amidation: (CO_2_H → CONH_2_)	Fungi, Bacteria, Yeast
	Buforin-2	-	Bacteria
Section 4.2	Goadsporin	Azole formation Dehydroalanine formation Leader peptide digestion N-acetylation	
	Nisin Z	Macrocyclization (thioether bond) Non-standard amino acids (e.g., *D*-alanine) Dehydroalanine, dehydrobutyric acid formation	*E. coli*, *B. subtilis*
Section 4.3	Salivaricin B	Dehydroalanine, dehydrobutyrine, and α-aminobutyric acid formation	Bacteria (*S. aureus RN4220)*
	Valinomycin	Non-standard amino acids (e.g., *D*-valine, *L*-lactate, *D*-hydroxyvalerate) Cyclic depsipeptide formation	Fungi, Gram-positive bacteria
	Gramicidin S	Non-standard amino acids (e.g., α-aminobutyric acid) Cyclic depsipeptide formation	Fungi, Bacteria
	*De novo* designed PBAs	-	*E. coli* (MG1655), *B. subtilis* (PY79), *E. faecium* *S. aureus* (DSM 11729), *K. pneumoniae* (DSM 30104), *A. baumannii*, *P. aeruginosa* (DSM 1117), *Enterobacter* spp., *Y. pestis* (EV76), *B. anthracis Sterne*, *S. pneumoniae* (D39)
	Meucin-25	-	Fungi, Bacteria
	Cathelicidin-BF	-	Fungi, Bacteria
	δ-lysin	-	Gram-positive bacteria
Section 4.4	SFTI-1	Disulfide bond formation	Fungi
	Kalata B1	Disulfide bond formation	Gram-positive bacteria
	AA139	Disulfide bond formation	Gram-negative bacteria
	HT-1	Disulfide bond formation	Bacteria
	Capistruin	Macrocyclization Leader peptide digestion	Gram-negative bacteria
	Thiocillin	Dehydroalanine formation Cycloaddition	Fungi, Gram-positive bacteria

## 5. Concluding Remarks

In this review, we have briefly highlighted several key aspects that, in our view, represent the recent major trends in the development of PBAs (Table 5). We acknowledge that our perspective is influenced by our research interests, and we recognize that some significant milestones in the field may not have been covered. However, our primary goal was to convey the rapid and extensive progress driven by CFSs in the area of PBAs, an area we believe not only lacks comprehensive summaries but also requires further research. Given that CFSs significantly contribute to the advancement of synthetic biology, particularly through their ability to rapidly prototype and screen ideal candidates in a high-throughput manner, this capability is poised to facilitate the identification and optimization of biosynthetic pathways for the effective synthesis of PBAs. Additionally, new chemo-enzymatic reactions tailored to these pathways have been developed, which enabled the successful synthesis of diverse PBAs that were previously unattainable through traditional methods with unique structures and biological properties. These new PBAs are already being utilized in medical practice [44], and many are in the pipelines of several pharmaceutical companies, promising to sustain the momentum for the future.

A promising area of research involves developing PBAs that target multiple pathogens. Significant efforts have been made to create broad-spectrum PBAs. For instance, some studies have focused on designing PBAs that target bacterial membranes, which, despite species-specific differences, share common structural features that can be exploited. However, the effectiveness of membrane-targeting PBAs can be limited by the variability in membrane compositions across different bacterial species. More recent research has shifted towards PBAs that bypass the membrane entirely, targeting intracellular components universally conserved among bacteria, such as ribosomes [63,148,156,207,208] or specific enzymes [209] involved in essential processes like DNA replication [15] or cell wall synthesis [210]. One notable example is the development of antimicrobial peptides that can permeate bacterial membranes and specifically target the bacterial ribosome [211], potentially inhibiting protein synthesis in a manner similar to that of tetracycline, but with broader efficacy against multiple pathogens.

The combination of computational approaches may significantly accelerate the discovery of PBA candidates that meet the desired criteria. Specifically, computational tools can be used to analyze large datasets of peptide sequences, structures, and antimicrobial activities to identify patterns associated with broad-spectrum efficacy. Machine learning models can predict new peptide sequences likely to be effective against multiple pathogens based on existing data. These predictions can then guide the design of de novo peptides, which can be synthesized and tested using CFSs.

Despite the potential of computational models, several challenges remain. For example, ncAAs predicted by computational methods must be both chemically synthesizable and compatible with the wild-type translational machinery or NRPSs. Moreover, if these ncAAs are not accepted by the cellular system, it may be necessary to engineer components of the translational machinery, such as aaRSs, tRNAs, elongation factors, and ribosomes. Additionally, ensuring that PBAs can effectively reach and interact with intracellular targets across different pathogens remains a critical hurdle. In the long-term perspective, these issues can be addressed through the principles of synthetic biology; specifically, the design–build–test–learn approach, particularly when applied within CFSs. These systems provide a flexible platform to refine PBAs for improved intracellular delivery and targeted interaction. Ultimately, we anticipate that integrating these advanced technologies will create a powerful platform for discovering new broad-spectrum PBAs, which are critically needed to address the growing issue of antibiotic resistance. Moreover, these studies will also deepen our understanding of protein synthesis and contribute valuable tools to the field of synthetic biology.

## Figures and Tables

**Table 1:** Characteristics of various PBAs and their activity against various pathogens.

Type	Peptide	Proposed Antibacterial Mechanisms	Target Gram-Negative Pathogens and MIC	Target Gram-Positive Pathogens and MIC	MIC for MDR
Peptides	Magainin II [19,20]	Membrane disruption	*E. coli* DH5α (3.125 μM) *P. aeruginosa* (12.5 μM)	*S. aureus* (100 μM)	MR *P. aeruginosa* (12.5 μM) MR *S. aureus* (8–64 μg/mL)
	Melittin [21,22,23]	Membrane disruption	*P. aeruginosa* (25–50 μg/mL)	*S. saprophyticus* ATCC 15305 (1 μM) *S. haemoliticus* (1 μg/mL)	*A. baumanni* (31 μg/mL) MR *S. aureus* (0.12 μM)
	LL-37 [24,25,26]	Membrane disruption Immunomodulation	*E. coli* ATCC 11775 (1.9 μg/mL)	*A. baumannii*-ATCC 19606 (32 μg/mL)	MR *A. baumannii* (16–32 µg/mL) MR *P. aeruginosa* (32–287.5 μg/mL)
	Buforin II [20,27]	Membrane disruption Intracellular targeting (nucleic acid)	*E. coli* (0.125–2 μg/mL)	*P. aeruginosa* (1–16 μg/mL)	*A. baumanni* (8–16 µM) MR *S. aureus* (1–8 μg/mL)
	Cecropin P1 [20]	Membrane disruption	*E. coli* (0.125–2 μg/mL)	*P. aeruginosa* (2–32 μg/mL)	MR *S. aureus* (16–64 μg/mL)
	Indolicidin [20]	Membrane disruption Intracellular targeting (inhibition of DNA replication and transcription, Ca^2+^-calmodulin interaction)	*E. coli* (0.50–8 μg/mL)	*P. aeruginosa* (4–64 μg/mL)	MR *S. aureus* (2–16 μg/mL)
	PR-39 [23,28]	Intracellular targeting (nucleic acid)	*E. coli* K12 (0.3 µM)	*S. aureus* (200 µM)	MR *S. aureus* (>40 μM)
	Vancomycin [29,30]	Inhibition of cell wall synthesis	-	*S. aureus* (4–8 μg/mL)	MR *S. aureus* (≤2 μg/mL)
	Daptomycin [23,31]	Membrane disruption	-	*S. aureus* (0.25–1 μg/mL)	MR *S. aureus* (0.125–1.0 μg/mL)
	Polymyxin [32,33]	Membrane disruption	*P. aeruginosa* (≤ 2 μg/mL)	*A.* spp (≥4 μg/mL)	*A. baumannii* 3367 (0.25 μg/mL) *A. baumannii* 100 (8 μg/mL)
Small molecules	Ciprofloxacin [27,34]	Intracellular targeting (DNA gyrase)	*E. coli* (0.004–0.25 μg/mL)	*E. faecium* (0.8–25 μg/mL)	*A. baumanni* (128 µM)
	Gentamicin [27,35]	Intracellular targeting (ribosome)	*E. coli* (4 μg/mL)	*E. species* (1–16 µg/mL)	*A. baumanni* (256 µM)
	Tetracycline [36,37]	Intracellular targeting (ribosome)	*E. coli* K12 TB1 (2–16 μg/mL)	*P. aeruginosa* (16–32 µg/mL)	*A. baumannii* (>128 μM)

**Table 3:** Four major PTMs and their reaction products.

Types of PTMs	PTM Reactions		PBAs	
Cyclization	Bridging reactions	Thioether formation	Nisin A Thuricin H	Subtilosin A
		Disulfide bond formation (Cysteine oxidation)	Apamin Kalata B1	Bactenecin
	Head-to-tail cyclization		Circularin A Microcystin-LA Subtilosin A	Kalata B1 Patellamide A
Cyclodehydration	Heterocycle formation		Goadsporin Thiostrepton	Patellamide A TP-1161
Alkylation	N-methylation		Cyclosporin A Thiostrepton	Microcystin-LA
Glycosylation	N-Glycosylation		Eosinophil cationic protein (ECP)	
			h-LF Tyrocidine A	Lactoferrin
	S-Glycosylation		Sublancin	
	O-glycosylation		Datucin Diptericin	Formaecin Drosocin

**Table 4:** Main research topics on PBAs using CFSs.

	Mechanisms (Section 4.1)	Biosynthetic Pathways (Section 4.2)	Selections (Section 4.3)	Synthesis (Section 4.4)
Methods	Mutagenesis of PBAs [148]	Identification of biosynthetic pathways [149,150,151]	Screening of multiple variants [139,152]	Inducing proper folding [153,154]
	Qualitative and quantitative analysis on biomolecules [16,63,155,156]	Enzyme promiscuity for substrates [157,158,159,160,161,162,163]	Optimization of reaction conditions [164,165,166]	Chemo-enzymatic modification [167]
		Kinetic analysis of enzymes [152]	Integration of deep generative models [168,169]	
			Generation of diverse environments through microfluidic system [170]	
			Target peptide selection using mRNA displays [171,172]	
